# Online and school bullying roles: are bully-victims more vulnerable in nonsuicidal self-injury and in psychological symptoms than bullies and victims?

**DOI:** 10.1186/s12888-023-05341-3

**Published:** 2023-12-14

**Authors:** Boglárka Drubina, Gyöngyi Kökönyei, Dóra Várnai, Melinda Reinhardt

**Affiliations:** 1 Doctoral School of Psychology, ELTE Eötvös Loránd University, Budapest, Hungary; 2 Institute of Psychology, ELTE Eötvös Loránd University, Budapest, Hungary; 3 NAP3.0-SE Neuropsychopharmacology Research Group, Hungarian Brain Research Program, Semmelweis University, Budapest, Hungary; 4 Department of Pharmacodynamics, Faculty of Pharmacy, Semmelweis University, Budapest, Hungary; 5 Heim Pál National Institute of Pediatrics, Budapest, Hungary; 6 14th District Medical Center, Child and Adolescent Psychiatry, Budapest, Hungary

**Keywords:** School bullying roles, Online bullying roles, Nonsuicidal self-injury, Externalizing problems, Internalizing problems, Adolescents

## Abstract

**Background:**

Bullying leads to adverse mental health outcomes and it has also been linked to nonsuicidal self-injury (NSSI) in community adolescents. It is not clear whether different roles of bullying (bully, victim, bully-victim) are associated with NSSI, furthermore the same associations in cyberbullying are even less investigated.

**Methods:**

The aim of the current study was to test whether students involved in school or online bullying differed from their not involved peers and from each other in psychological symptoms (externalizing and internalizing problems) and in NSSI severity (number of episodes, number of methods). Furthermore, mediation models were tested to explore the possible role of externalizing and internalizing problems in the association of school and online bullying roles with NSSI. In our study, 1011 high school students (66.07% girls; n = 668), aged between 14 and 20 years (M_age_ = 16.81; SD = 1.41) participated.

**Results:**

Lifetime prevalence of at least one episode of NSSI was 41.05% (n = 415). Students involved in bullying used more methods of NSSI than not involved adolescents. In general, victim status was associated mostly with internalizing symptoms, while bully role was more strongly associated with externalizing problems. Bully-victims status was associated with both types of psychological problems, but this group did not show a significantly elevated NSSI severity compared to other bullying roles. Externalizing and internalizing problems mediated the relationship between bullying roles and NSSI with different paths at different roles, especially in case of current NSSI that happened in the previous month.

**Conclusions:**

Results highlight that students involved in bullying are more vulnerable to NSSI and to psychological symptoms compared to their peers who are not involved in bullying. It is suggested that bullying roles, especially bully-victim status, need to be identified in school and online settings and thus special attention should be addressed to them to reduce psychological symptoms and NSSI, for example by enhancing adaptive coping skills.

**Supplementary Information:**

The online version contains supplementary material available at 10.1186/s12888-023-05341-3.

## Introduction

As nonsuicidal self-injury (NSSI) – the intentional, direct destruction (e.g., scratching, bruising, cutting, burning, biting) of one’s own body tissue without suicidal intent [1] – is a considerable behavioral problem, especially among adolescents [2, 3], and has become an even more widespread phenomenon throughout the last decade [4, 5], research should focus more on this topic. Some evidence indicates that NSSI can be a stronger predictor of suicide attempts than previous suicidal behavior [6, 7]. NSSI typically occurs in early adolescence (between the age of 12–14) [5, 8] and peaks in mid-adolescence [9, 10]. The prevalence can be remarkably high in community youth samples (between 14.5 and 46.5%) [11–13], and based on meta-analytical results, females are more at risk for NSSI [14].

The current study focuses on three phenomena typically occuring during adolescence and thus may also be the cause of difficulties in school settings: along with *NSSI*, we also focus on *bullying*, and *internalizing* and *externalizing* problems. While previous research mainly focuses on bullying victimization and victims, by differentiating the roles of bullying (bully, victim, bully-victim), the aim of this study is to investigate whether bullies and bully-victims are also vulnerable to mental health issues (NSSI, internalizing and externalizing problems). Furthermore, another intention of this study is to establish and test mediation models that can be taken into consideration when planning NSSI and mental health related interventions in school settings. Our research includes cyberbullying as well, which is less investigated as school-based bullying but more and more a widely spread type of peer aggression.

### Bullying and bullying roles

In the current study, bullying is defined as a type of youth violence which includes any unwanted aggressive behavior by another youth or group of youths who are not siblings or current dating partners [15, 16]. Bullying can take place in the school or in any online platforms, the latter is cyberbullying [17, 18]. In our study, a traditional classification of bullying roles was applied: perpetrator, victim, and bully-victim [19, 20]. *Bully/perpetrator* is the person who commits the bullying and have a perceived dominance or more power than the victim. *Victim* is a person who suffers from being bullied and perceived as less dominant or having less power. B*ully-victim*s are those who are both victims and perpetrators [19–21].

Regarding bullying roles, gender differences can be observed: boys are more likely to engage in bullying others in school and in online settings [22, 23], while girls are more likely to be the victims of online bullying [24]. Based on the data of the HBSC (Health Behavior in School-aged Children) study obtained in 2017/18, the prevalence of bullying perpetration and victimization shows a great variety across the 45 participating countries (the prevalence of perpetration varied from 0.3 to 30% in the 11–15-year-old students, and victimization rates ranged from 0.5 to 32%, respectively) [25]. However, in many countries there is a decline in bullying perpetration rates. Similar to offline bullying, a significant cross-national variety of prevalence regarding cyberbullying is observable (from 0.6 to 31% in cyberbullying others and from 3 to 29% in being cyberbullied) [25]. Regarding Hungary, according to the latest HBSC data collection, 28.4% of the 11–17-year-old students reported to have been bullied at least once or twice and 27.1% reported that they have bullied someone in school at least once or twice in the recent three months [26]. The rates of cyberbullying were lower: 17.8% of students have been cyberbullied and 12.7% of students bullied others online [26].

### The link between bullying roles and externalizing and internalizing problems

Based on different roles in traditional school-based bullying, previous research differentiates connected mental health problems in adolescents. Most of the studies report that bullying perpetrators are more likely to face externalizing problems, while victims are more likely to face internalizing problems [23, 27, 28]. However, the perpetrator-externalizing and victim-internalizing associations can be oversimplifying based on Cook and his colleagues [29] findings who included online bullying as well. In their meta-analytical results, internalizing problems can be associated with the bully role as well (effect size = 0.12), but the association is stronger for the victims (effect size = 0.25). And similarly, externalizing problems are significantly associated with the victim role (effect size = 0.12), but the association is stronger for bullies, making externalizing problem behaviors the strongest individual predictor of being a bully (effect size = 0.34). Bully-victim role was associated with both externalizing (effect size = 0.33) and internalizing (effect size = 0.22) problems [29]. Although, previous studies have demonstrated that being a bully-victim in traditional school-based bullying [22, 30–32] or in cyberbullying [33, 34] might be associated with worse mental health outcomes than either bullies or victims, only a few studies investigated the characteristics and mental health problems of this vulnerable group [25].

### The link between bullying and NSSI

Several risk factors of NSSI have been suggested in previous research (e.g., emotion regulation problems, impulsivity, depressive symptoms), that are mainly individual characteristics [35]. Less interest has been given to school and peer factors, although the climate of peer relationships or related negative life events can also play an important role in the development of NSSI, furthermore, as it has been recently suggested, academic-related stress and peer bullying is associated with NSSI behaviors [36], therefore investigating bullying in association with NSSI is essential.

Many cross-sectional studies suggest that adolescents who reported being victims of bullying were at an increased risk for NSSI compared to adolescents who were not victims of bullying or who reported low levels of victimization [37–39]. Based on two recent studies, involvement in bullying increases the likelihood to engage in NSSI [40, 41]. Meta-analytical findings also suggest that bullying is associated with NSSI [14, 42]. However, not many studies focused on perpetrators or bully-victims, the associations with NSSI of these roles or the trajectories through which perpetrators or bully-victims are linked to NSSI. Some results show that bullies also engage in NSSI [43], especially when they had a history of being bullied (which could have made them bully-victims) [22, 44]. Additionally, bully-victim girls scored the highest in NSSI [44] compared to any other role of bullying, and NSSI has also been linked to cyberbullying [45]. Only a very limited number of research has focused on the association between NSSI and cyberbullying, and these studies mainly investigated cyberbullying victimization. Recent research however shows a higher frequency of NSSI among students involved in cyberbullying and shows that cyberbullying can be in direct association with NSSI [12, 46, 47]. Although online and school-based bullying share common features (e.g., bullying roles, association with mental health problems) remarkable differences occur as well (e.g., higher anonymity, fewer intervention opportunities for affected students, loneliness, role of internet safety features) [18]. Being alone in online settings might facilitate the appearance of NSSI as feeling lonely is associated with NSSI [48, 49] and NSSI happens most often when adolescents are alone [50]. Furthermore, compared to school-based bullying, online bullying might be more difficult to deal with for the environment (e.g., parents, educators) [51], making it more difficult for the child to cope with it in the lack of adequate help from significant ones which can also result in a maladaptive coping strategy (e.g., NSSI). A longitudinal study found that cyberbullying can cause harm above and beyond traditional bullying [52]. Therefore, regarding the remarkable differences between cyberbullying and school-based bullying, it is essential to be able to compare whether bullying in different settings have the same association to NSSI or not. In the current study, online and school-based bullying roles are tested with different mediation models.

Although, most of the previously mentioned findings are cross sectional, some longitudinal cohort and case control studies [14, 44] suggest that bullying is not only associated to NSSI but may also predict it.

### The link between externalizing and internalizing problems and NSSI

NSSI is often considered as a transdiagnostic element in psychopathology, therefore NSSI-related variables (e.g., suicidality or impulse control difficulties) are best predicted by transdiagnostic variables [53]. NSSI episodes are prevalent in different psychiatric disorders and psychological symptoms during adolescence (e.g., depression, psychotic symptoms, substance abuse, borderline personality-disorder features, conduct problems, emotional problems) [54, 55] and has been linked to externalizing problems (e.g., attention deficit hyperactivity disorder, conduct disorder, oppositional defiant disorder) [56], and to internalizing problems as well in adolescence [57]. Some findings suggest that externalizing and internalizing psychopathology are not only associated, but longitudinally predict NSSI [58].

### Externalizing and internalizing problems as possible mediators

Bullying victimization and perpetration can result in interpersonal difficulties or negative interpersonal life events that can cause stress, negative emotions, and mental health problems in adolescence [59–64]. Subsequently, mental health problems and interpersonal difficulties can trigger and might result in NSSI, thus, they occur comorbidly [65, 66]. To cope with negative emotions, NSSI might appear as a dysfunctional emotion regulation [3, 67, 68].

As described in the vulnerability-stress model, suggested by Hankin and Abela [69], internalizing and externalizing problems are rooted in both individual factors (cognitive vulnerabilities) and in environmental factors (stressors, negative life events, adversities). Environmental factors can be adverse life events that might strengthen the possibility of the development of mental health problems, psychopathology and NSSI in adolescents [32, 70]. Bullying might be a significant environmental factor as throughout adolescence the importance of peer relationships and their opinion on oneself can considerably increase the negative influence on the quality of mental health [71, 72]. Few mediator models have been established to explain the relationship between bullying and NSSI. Researchers so far have found a partial mediation regarding negative emotions [45], depressive mood and depressive symptoms [22, 37]. In the current study, the possible mediating effect of internalizing and externalizing problems on the relationship between bullying and NSSI among community adolescents was hypothesized and tested.

### Aim of the study

The global aim of the current study is to investigate the relationship of school and online bullying, externalizing and internalizing problems, and NSSI.

By establishing different bullying roles, this study seeks answers to the question whether those students who are involved in bullying suffer from greater mental health problems compared to students who are not involved in bullying. The following hypotheses were established:

1. Adolescents involved in (school or online) bullying show significantly higher internalizing and externalizing scores compared to not involved peers. It is also hypothesized that bully-victims score significantly higher in internalizing and externalizing problems compared to any other peer group (bullies, victims, not involved adolescents).
2. Adolescents involved in (school or online) bullying show significantly more serious NSSI behavior (in terms of the number of NSSI episodes and the number of NSSI methods) compared to not involved peers. It is also hypothesized that bully-victims show significantly more serious NSSI behavior compared to any other peer group (bullies, victims, not involved adolescents).

**Fig. 1:**
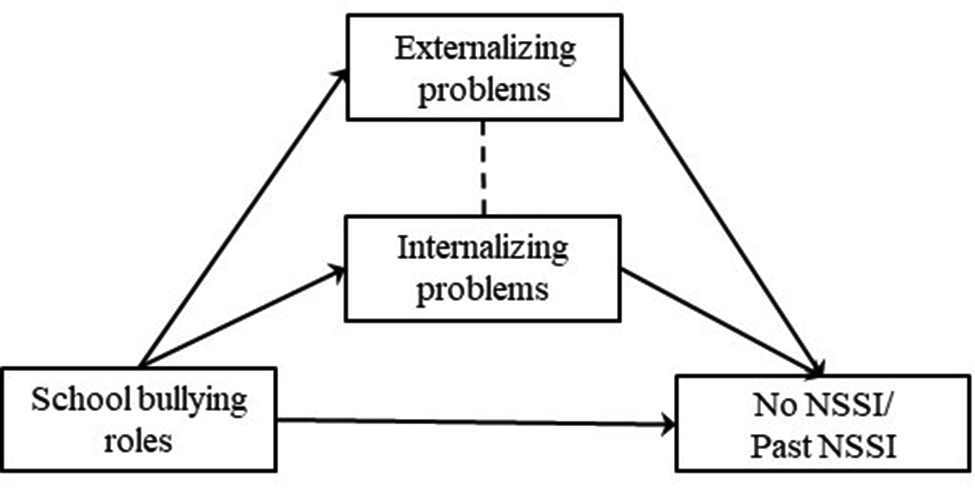
Hypothesized mediation model 1

**Fig. 2:**
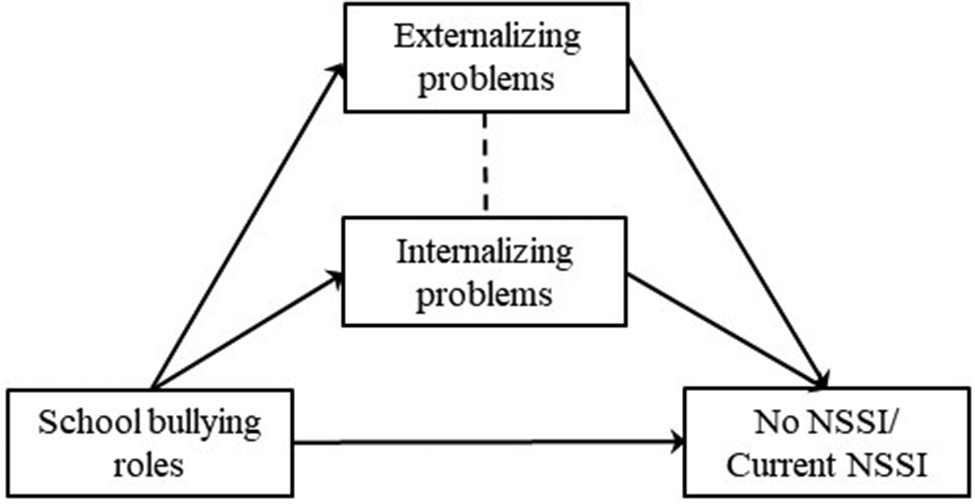
Hypothesized mediation model 2

**Fig. 3:**
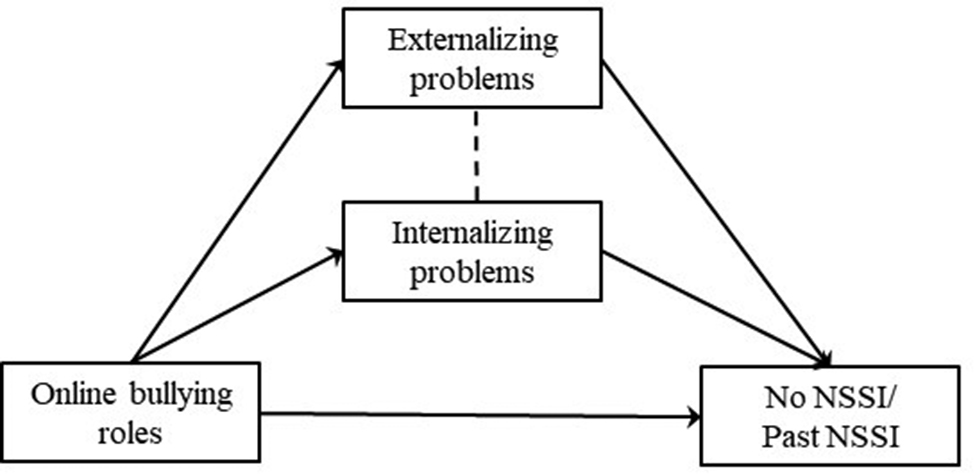
Hypothesized mediation model 3

**Fig. 4:**
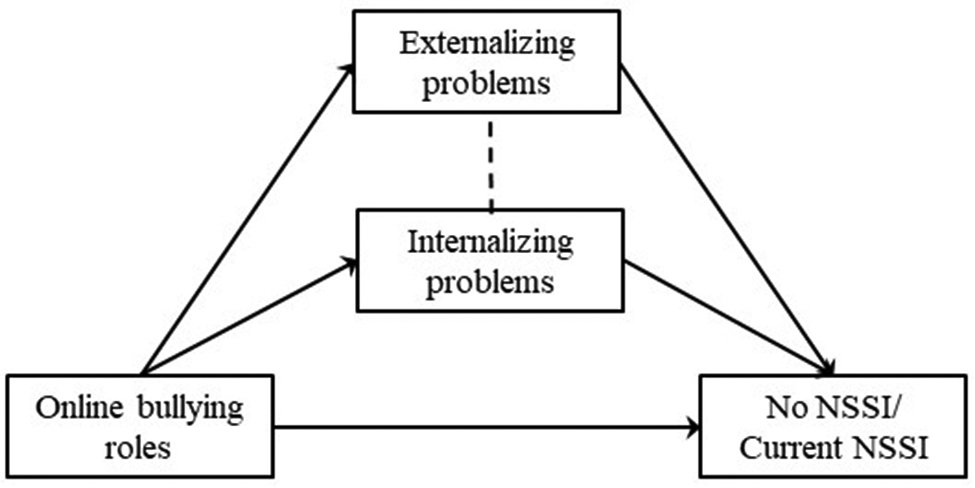
Hypothesized mediation model 4

**Fig. 5:**
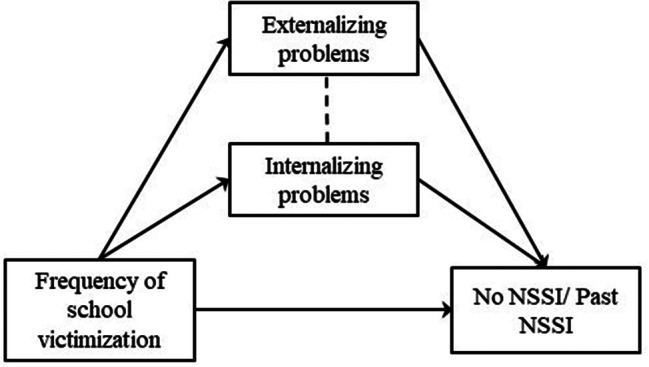
Hypothesized mediation model 5

**Fig. 6:**
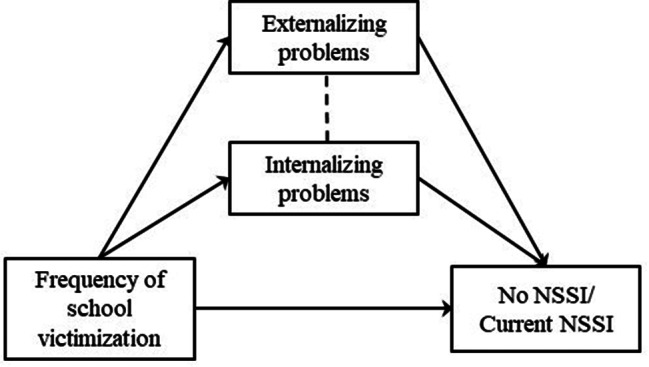
Hypothesized mediation model 6

Considering that externalizing and internalizing problems are both correlated to bullying [23] and NSSI [65], the current study establishes six mediation models (Figs. 1, 2, 3, 4, 5 and 6) to understand the relationship among different school and online bullying roles, externalizing and internalizing problems, and NSSI. Accordingly, the following hypotheses were examined:

(3) The association of school and online bullying roles with NSSI will be partially mediated by both externalizing and internalizing symptoms (Figs. 1, 2, 3 and 4) given that both bullying perpetration and victimization might be related to internalizing and externalizing problems [29];
(4) The association of the frequency of school victimization and NSSI will be partially mediated by externalizing and internalizing problems (Figs. 5 and 6, respectively).
  Gender and age are taken into consideration as control variables in the mediation models. As NSSI is predicted by various factors, it was hypothesized that externalizing and internalizing problems would only decrease the direct effect of bullying on NSSI rather than eliminate the association. The study also seeks answer to the question whether bully-victims are in need of intervention due to higher mental health problems (i.e., NSSI, externalizing and internalizing problems) compared to victims and bullies and not involved students.

## Materials and methods

### Participants and procedure

This cross-sectional study involved 14 Hungarian secondary schools from the capital city and from the countryside. Specific schools were asked to participate based on accessibility to the researchers while following the idea to represent different type of secondary schools based on location (e.g., metropolitan area, smaller cities) and on educational profile (e.g., high-school, vocation schools). Data collection started in February 2019 and finished in January 2020. Participants were from all grades (grades 9–12) of secondary schools. During data collection, trained investigators were present, but not any teachers. Students filled out the questionnaires either in their classroom (paper-based questionnaires) or online (on the Qualtrics platform) in computer rooms or on smart phones according to the circumstances of the schools. Online questionnaires were filled out in the classroom, in-person settings as well (e.g., during informatics class) in the presence of trained investigators.

More than one thousand and two hundred students (N = 1232) were requested to take part in the study and a total of 1059 students agreed in participating. 173 students were either absent during data collection or declined to participate. From the 1059 who agreed in the participation, 48 were excluded due to incomplete answers. Thus, the final sample consists of 1011 students, mostly females (n = 668; 66.07% girls), the mean age came to 16.81 (SD = 1.41) years. The youngest participants were 14 years old; the oldest participants were 20 years old. Most of the participants live in cities (n = 450, 44.5%) or in the capital (n = 252; 24.9%), while 309 (30.6%) students live in villages.

All aspects of the study were ethically approved by the Institutional Review Board of ELTE Eötvös Loránd University, Budapest, Hungary. Participation was voluntary and anonymous. Students and one of their parents had to give their written consent to participate in the research, while headmasters of secondary schools were also informed about the details of the study and gave their consent to carry out the research in their institution. The Declaration of Helsinki [73] was taken into account while carrying out the research. An information sheet about the meaning, characteristics of NSSI and possible sources (online and in person) of help was provided to every participant.

### Measures

#### Inventory of Statements About Self-injury

NSSI is often measured with the Inventory of Statements About Self-Injury (ISAS) [74]. In this study, Hungarian version of the short form was used [75] (Hungarian version: [76]). The short form of ISAS has two parts, the first assesses prevalence, types (12 different – plus one free answer – NSSI behaviors, e.g., cutting, biting, carving, severe scratching or hitting self) and characteristics of NSSI (e.g., age of onset, the experience of pain during NSSI, whether NSSI is performed alone or around others). The second part measures 13 functions of NSSI [74]. In the current study, only frequency, methods of NSSI and time of the last episode were analyzed from the first part of the ISAS. At the beginning of the questionnaire, definition of NSSI was given, underlying the importance of the act being deliberate without suicidal intent.

#### Bullying

The Hungarian version of the Revised Olweus Bully/Victim Questionnaire [77] was used to measure bullying which was used in the Hungarian HBSC Study [26]. First, a precise definition was given to participants about the meaning of bullying: “We say a student is being bullied when another student, or several other students say mean and hurtful things to him or her; or make fun of him or her; or call him or her mean and hurtful names; or completely ignore or exclude him or her from their group of friends or leave him or her out of things on purpose. When we talk about bullying, these things happen repeatedly, and it is difficult for the student being bullied to defend himself or herself. We also call it bullying, when a student is teased repeatedly in a mean and hurtful way. But we don’t call it bullying when the teasing is done in a friendly and playful way. Also, it is not bullying when two students of about equal strength or power argue or fight.” In the first part, school bullying was measured with two questions: the first measures the frequency of bully perpetration during the past few months, while the second asks about the frequency of bully victimization during the same period of time. Online bullying was measured in the same way. The definition of online bullying was also given before the questions, containing examples as well (e.g., sending offensive messages to someone via SMS, chat programs or e-mail, posting such message on someone’s wall on social media).

Based on the previous questions, four different roles of school and online bullying were differentiated: *school bullies* were participants who at least once or twice have bullied someone else at school during the previous months, but they have not been bullied at all at school. *Online bullies* were participants who at least once or twice have bullied someone else online during the previous months, but they have not been bullied online. *School victims* were those participants who have been bullied at least once or twice during the previous months at school, but they have not bullied others at all in school settings. *Online victims* were those participants who have been bullied at least once or twice during the previous months on online platforms, but they have not bullied others at all online. *School bully-victims* are those participants who have bullied others at least once or twice at school in the previous months and who have been bullied as well at least once or twice at school during the previous months. *Online bully-victims* are those participants who have bullied others at least once or twice online in the previous months and who have been bullied as well at least once or twice online during the previous months. *Not involved* students have not bullied others and have not been bullied either, neither in school, nor online during the previous months.

In the second part of the questionnaire, seven different types of school bullying victimization (e.g., being excluded from activities or social groups, being ignored, being mocked) were measured. Items 6 (being mocked because of religion) and 7 (experiencing sexual comments) were developed by the Canadian HBSC group [78].

Participants could give their answers on a 5-point Likert-scale (1 = *never during the past few months*, 2 = *once or twice*, 3 = *two or three times a month*, 4 = *approximately once a week*, 5 = *several times a week*).

In the current study, reliability of the second part of the questionnaire (types of school victimization) was good (α = 0.70). Previous studies did not report reliability data concerning the second part of the Revised Olweus Bully/Victim Questionnaire [77].

#### Strengths and Difficulties Questionnaire (SDQ)

The self-report version of the Strengths and Difficulties Questionnaire [79] (Hungarian translation: [80]) is a brief emotional and behavioral screening questionnaire for children and young people and is a valid and reliable instrument to measure externalizing and internalizing symptoms in adolescence. Respondents use a 3-point scale to indicate how far each item applies to them (1 = *not true*, 2 = *somewhat true*, 3 = *completely true*). The 25 items are divided between 5 subscales, with five items: emotional symptoms (e.g., “*I am often unhappy, downhearted or tearful.*”), conduct problems (e.g., “*I fight a lot. I can make other people do what I want.”*), hyperactivity-inattention (e.g., “*I am constantly fidgeting or squirming.”*), peer problems (e.g., “*I am usually on my own. I generally play alone or keep to myself.”)*, and prosocial behavior (e.g., *“I often volunteer to help others (parents, teachers, children).”*). The authors of the questionnaire suggest the use of a three-subscale division of the SDQ [81] in low-risk or general population samples: *internalizing problems* (emotional symptoms + peer problems, 10 items), *externalizing problems* (conduct problems + hyperactivity symptoms, 10 items) and prosocial scale (5 items). In the current study, only internalizing and externalizing subscales were used. Reliability of the subscales were good in our study (internalizing subscale α = 0.75 was better than the original’s α = 0.66; externalizing subscale α = 0.76 was similar to the one in the original study α = 0.76) [81].

### Data analysis

Basic characteristics of the sample, descriptive statistics of the variables and correlations were performed in IBM SPSS 28, the level of significance was taken as 0.05. Mediation analyses were performed in Mplus 8.0 [82].

NSSI was analyzed via two binary variables in the mediation models: one consists of *no history of NSSI* and *past NSSI* (at least one NSSI episode in the past, earlier than a month), while the other consists of *no history of NSSI* and *current NSSI* (at least one NSSI episode in the last month). In the ANOVA analysis, NSSI was a categorical variable with three values: no history of NSSI, past NSSI and current NSSI.

Two variables measured the severity of NSSI. Number of NSSI methods was a continuous variable, while number of NSSI episodes was a binary variable (non-repetitive NSSI = 1–9 episodes, repetitive NSSI = 10 or more episodes based on the suggestion of Gratz et al., [83]).

In the case of bullying, three different bullying variables were analyzed: (1) *school bullying roles* (1 = victim, 2 = bully, 3 = bully-victim, 4 = not involved in bullying) as a categorical variable; (2) *online bullying roles* (categories are the same as at school bullying); and (3) *frequency of different school victimization types* (higher score means more frequent school bullying victimization; used as a continuous variable).

Victim status was measured in two ways: in the first part of the questionnaire, the frequency of bullying victimization was asked in general (“*How often were you bullied during the past months?”*), while in the second part concrete items measure the frequency of different school bullying types.

As suggested in the literature [84], dummy variables were created for independent categorical variables (school and online bullying roles, see categories in the *Measures* part) to use in mediation modeling. Externalizing and internalizing problems were continuous variables, higher scores mean stronger internalizing and externalizing problems.

Pearson correlations were performed to measure associations between different variables. Group differences (NSSI, school bullying roles, online bullying roles) regarding externalizing and internalizing problems, and NSSI severity (number of NSSI methods) were assessed with one-way ANOVA. Crosstabulation was performed regarding the number of NSSI episodes (binary variable). In the case of NSSI severity (number of NSSI methods and number of NSSI episodes) normal distribution was violated, therefore the robust version of ANOVA (Welch test) was used. When Levene’s test claimed the violation of the homogeneity of variances, a robust post-hoc test (Games-Howell) was used, otherwise, the results of non-robust post-hoc test (Tukey) are reported. Post-hoc tests are used to compare group differences. To avoid type 1 error, p-values were adjusted during post-hoc analyses in the following way: when analyzing NSSI severity and internalizing and externalizing symptoms in different school and online bullying groups, 4 groups were compared with each other that resulted in 6 comparisons (not involved students vs. bully-victims; not involved students vs. bullies; not involved students vs. victims; bully-victims vs. bullies; bully-victims vs. victims; bullies vs. victims), therefore, p value of 0.05 was divided by 6 which results in p < .0083. Comparing three different NSSI groups in regards with internalizing and externalizing symptoms, three comparisons were made (no NSSI vs. past NSSI; no NSSI vs. current NSSI; past NSSI vs. current NSSI) therefore p value of 0.05 was divided by 3 which results in p < .016. In the Results, only results significant according to the adjusted level are reported. In the comparisons of groups, reference group was the not involved students in case of bullying roles. Regarding NSSI, reference was the no NSSI group (students who have never engaged in NSSI).

Six mediation models were established based on the literature and tested in the current study via Structural Equation Modeling. Mediator variables in every model were both externalizing and internalizing problems. Due to low correlation between the two mediator variables (r = .27), they were tested parallelly within the same models. Gender (1 = boys, 2 = girls) and age (continuous variable) were control variables in each mediation model.

In the models, observed variables were school bullying roles (Model 1, Fig. 1), online bullying roles (Model 2, Fig. 2) and frequency of school victimization (Model 3, Fig. 3). Outcome variables were No NSSI/Past NSSI and No NSSI/Current NSSI. Due to categorical variables, MLR (robust version of maximum likelihood parameter) estimator was used to perform Structural Equation Modeling [85]. Models are saturated as every possible connection is coded in the models.

## Results

### Characteristics of NSSI

Almost one third (n = 320, 31.7%) of the current sample engaged in NSSI at least once within the last month. Prevalence of past NSSI was 9.4% (n = 95; participants who had engaged in NSSI some point in their life but did not engage within the last month). Among those who have ever engaged in NSSI, the most common methods were banging or hitting oneself (n = 222; 53.1%), interfering with wound healing (n = 218; 52.2%), cutting (n = 170; 40.7%), biting (n = 163; 39%), pinching (n = 162; 38.8%) and severe scratching (n = 144; 34.4%). Mean age of the first NSSI episode was 11.99 years (SD = 3.52). Significantly more girls (n = 293; 43.86%) engaged in NSSI than boys (n = 121; 35.27%; χ^2^(1) = 32.40; p < .001). The highest number of used NSSI methods was 11 from 13. Among those who have engaged in NSSI, 3.80 (SD = 2.69) methods were used in average. Mean score for number of NSSI episodes was 111.91 (SD = 935.96). Lifetime repetitive NSSI (≥ 10 episodes based on the suggestion of Gratz [83]) was 72.05% (n = 299) of those who have ever engaged in NSSI.

### Descriptive statistics and correlation of the variables

**Table 1:** Descriptive statistics and correlation of variables

	*Min.*	*Max.*	*M*	*SD*	1.	2.	3.	4.	5.	6.	7.
1. gender^a^											
2. age	14	20	16.81	1.42	0.03						
3. SCH victimization	0	28	1.74	2.75	− 0.06^*^	− 0.08^**^					
4. SDQ-externalizing	0	18	6.61	3.11	− 0.04	0.03	0.27^***^				
5. SDQ-internalizing	0	18	6.61	3.75	0.29^***^	0.07^*^	0.29^***^	0.27^***^			
6. Number of NSSI methods	0	11	1.68	2.55	0.08^**^	0.01	0.33^***^	0.28^***^	0.34^***^		

*Note*. ***p < .001; **p < .01; *p < .05; a: 1 = female, 2 = male; SCH victimization = frequency of school victimization; SDQ-externalizing = externalizing score of the Strengths and Difficulties Questionnaire; SDQ-internalizing = internalizing score of the Strengths and Difficulties Questionnaire.

Descriptive statistics and correlation of continuous variables, gender and age are shown in Table 1. Female gender was associated to increased internalizing problems and to number of NSSI methods. Frequency of different school victimization types was slightly associated to lower age, while higher level of internalizing problems was associated to being older. School victimization was almost equally associated to internalizing and externalizing problems. Higher number of used NSSI methods was associated to a higher level of internalizing, externalizing and to more frequent school victimization.

Table 2 shows the crosstabulation of number of participants involved in different roles of school and online bullying. More girls were victims of school bullying (χ^2^(1) = 6.12; p < .01), while significantly more boys were bullies (χ^2^(1) = 20.92; p < .01) and bully-victims (χ^2^(1) = 4.15; p < .05), compared to girls. Similar gender differences emerged in cyberbullying with higher number of female victims (χ^2^(1) = 4.62; p < .05), higher number of male bullies (χ^2^(1) = 10.06; p < .01), and bully-victims (χ^2^(1) = 8.39; p < .01). Most frequent school victimization types were being excluded from activities, social groups or being ignored (n = 344; 33.9%); spreading rumors or fake news (n = 292; 28.8%); being calling names and teasing or made fun of (n = 249; 24.5%) and being the target of sexual comments (n = 120; 11.8%). Those who have ever been or currently are involved in NSSI reported higher frequency of school victimization (M = 9.44; SD = 3.21) compared to peers who are not involved in NSSI (M = 8.24; SD = 2.25; t = 6.53; p < .001). The number of participants who were bullied at school in any form at least once or twice (n = 568) is considerably higher compared to those who reported being bullied at school (n = 91) or online (n = 93) when asking simply how frequently the bullying happened (no concrete types of bullying were given).

**Table 2:** Different school * online bullying roles crosstabulation

		Online bullying roles					
		Victim	Bully		Bully-victim	Not involved	Total^a^ n (%)
School bullying roles							
Victim	n	22	2		8	59	91 (9.01)
	% within school bullying	24.2	2.2		8.8	64.8	
	% within online bullying	23.7	6.5		17.0	7.0	
Bully	n	7	6		9	59	81 (8.01)
	% within school bullying	8.6	7.4		11.1	72.8	
	% within online bullying	7.5	19.4		19.1	7.0	
Bully-victim	n	16	3		11	35	65 (6.43)
	% within school bullying	24.6	4.6		16.9	53.8	
	% within online bullying	17.2	9.7		23.4	4.1	
Not involved	n	48	20		19	687	774 (76.55)
	% within school bullying	6.2	2.6		2.4	88.8	
	% within online bullying	51.6	64.5		40.4	81.9	
Total^a^ n (%)		93 (9.22)	31 (3.06)		47 (4.64)	840 (83.08)	

*Note*. a = % is based on N = 1011.

### Group differences in the severity of NSSI

Welch test revealed that different school-related bullying roles significantly differ in the number of NSSI methods (F (3;139.62) = 13.36; p < .001). Table 3 shows post hoc analysis and group differences. Those who have participated in school bullying in any form (bully, victim, bully-victim) use significantly more NSSI methods compared to their peers who have not participated in bullying at all. Similar results emerged in case of cyberbullying.

**Table 3:** Comparison of bullying roles in number of NSSI methods: post hoc test analysis

	n (%)	M(SD)	Mean difference			
*School bullying roles*			1.	2.	3.	4.
1.not involved	774 (76.55)	1.36(2.28)	-			
2.bully-victim	65 (6.43)	2.89(3.10)	**-1.53**	-		
3.bully	81 (8.01)	2.59(3.05)	**-1.22**	0.30	-	
4.victim	91 (9.00)	2.74(3.06)	**-1.38**	0.14	0.15	-
*Online bullying roles*						
1.not involved	840 (83.0)	1.41 (2.26)	-			
2.bully-victim	47 (4.65)	3.89(3.84)	**-2.48**	-		
3.bully	31 (3.06)	3.00(2.80)	**-1.59**	-0.89	-	
4.victim	93 (9.19)	2.62(3.16)	**-1.21**	1.26	0.37	-

*Note.* Significant results are marked with bold numbers.

A crosstabulation in Table 4 shows the rate of repetitive NSSI and non-repetitive NSSI in different bullying roles.

**Table 4:** Different school and online bullying roles * lifetime repetitive NSSI crosstabulation

		School bullying roles						
		Victim	Bully		Bully-victim	Not involved		Total^a^ n (%)
Lifetime repetitive NSSI								
Yes	n	41	35		35	188		299 (29.58)
	% within lifetime repetitive NSSI	13.7	11.7		11.7	62.9		
	% within role in school bullying	45.1	43.2		53.8	24.2		
No	n	50	46		30	586		712 (70.42)
	% within lifetime repetitive NSSI	7.0	6.4		4.2	82.4		
	% within role in school bullying	54.9	56.8		46.2	75.8		
		Online bullying roles						
		Victim	Bully		Bully-victim		Not involved	
Yes	n	36	16		25	222		299 (29.57)
	% within lifetime repetitive NSSI	12.0	5.4		8.4	74.2		
	% within online bullying	38.7	51.6		53.2	26.3		
No	n	57	15		22	618		712 (70.43)
	% within lifetime repetitive NSSI	8.0	2.1		3.1	86.9		
	% within online bullying	61.3	48.4		46.8	73.7		

*Note*. a = % is based on N = 1011.

### Group differences in externalizing and internalizing problems

One-way ANOVA revealed that NSSI groups significantly differ in both the level of externalizing problems (F(2,1007) = 26.11; p < .001) and internalizing problems (F(2,1007) = 62.28; p < .001) as well. Table 5 contains post hoc comparisons of different groups. Regarding externalizing symptoms, the mean score of no-NSSI group was significantly lower than the mean score of past NSSI group and current NSSI group. Internalizing problems showed the same pattern.

School and online bullying roles differed significantly in the level of externalizing problems (school bullying: F(3,1001) = 15.93; p < .001; online bullying: F(3,1003) = 17.88; p < .001) and in the level of internalizing problems as well (school bullying: F(3,1001) = 30.38; p < .001; online bullying: (F(3,1003) = 18.65; p < .001). Post hoc test revealed that every school bullying role showed significantly higher level of externalizing problems than those who were not involved in school bullying, with the highest average score of bully-victims and bullies.School bullying roles differed significantly in the level of internalizing problems as well. Compared to those who were not involved in bullying, victims and bully-victims had significantly higher scores of internalizing problems, but bullies did not differ significantly. Victims and bully-victims scored significantly higher on internalizing symptoms compared to bullies, but there was no significant difference between victims and bully-victims. Online bullying roles differed significantly in the level of externalizing problems. Post hoc test revealed that online bullies and online bully-victims scored significantly higher on externalizing problems than those who were not involved in online bullying.

Online bullying roles differed significantly in the level of internalizing problems as well. Post hoc test revealed that online victims and online bully-victims scored significantly higher on internalizing problems than those who were not involved in online bullying; regarding bullies there was no difference compared to the reference group.

**Table 5:** Comparison of bullying roles and NSSI groups in externalizing and internalizing problems: post hoc test analysis

		SDQ-externalizing						SDQ-internalizing					
	n(%)	M(SD)	Mean difference					M(SD)		Mean difference			
*NSSI*			1.		2.	3.				1.	2.	3.	
1.no	595 (58.96)	6.05(2.87)	-					5.59(3.30)		-			
2.past	95 (9.39)	6.87(2.95)	-0.82		-			7.16(3.46)		**-1.56**	-		
3.current	320 (31.65)	7.54(3.24)	**-1.48**		-0.66	-		8.30(3.97)		**-2.71**	**-1.14**	-	
*School bullying roles*				1.	2.	3.	4.		1.		2.	3.	4.
1.not involved	774 (76.55)	6.26(3.00)	-					6.13(3.55)		-			
2.bully-victim	65 (6.43)	8.34(3.11)	**-2.08**		-			8.52(3.58)		**-2.39**	-		
3.bully	81 (8.01)	7.79(3.07)	**-1.53**		0.54	-		6.29(3.44)		-0.16	**2.22**	-	
4.victim	91 (9.01)	7.20(2.92)	-0.94		1.13	0.59	-	9.51(4.08)		**-3.38**	-0.98	**-3.21**	-
*Online bullying roles*				1.	2.	3.	4.		1.		2.	3.	4.
1.not involved	840 (83.08)	6.34(2.99)	-					6.24(3.62)		-			
2.bully-victim	47 (4.64)	8.98(3.46)	**-2.64**		-			8.40(3.41)		**-2.16**	-		
3.bully	31 (3.06)	8.77(2.88)	**-2.42**		0.21	-		6.86(3.66)		-0.63	1.53	-	
4.victim	93 (9.22)	7.04(2.93)	-0.70		**1.93**	1.72	-	8.88(4.10)		**-2.64**	-0.47	-2.01	-

*Note.* Significant results are marked with bold numbers. SDQ-externalizing = externalizing score of the Strengths and Difficulties Questionnaire; SDQ-internalizing = internalizing score of the Strengths and Difficulties Questionnaire.

### Mediation analysis

All standardized regression coefficients and standard errors of total, direct, and indirect effects related to each model are detailed in the Supplementary Materials Table S1. While Table S2 in the Supplementary Materials contains odds ratios and confidence intervals.

**Fig. 7:**
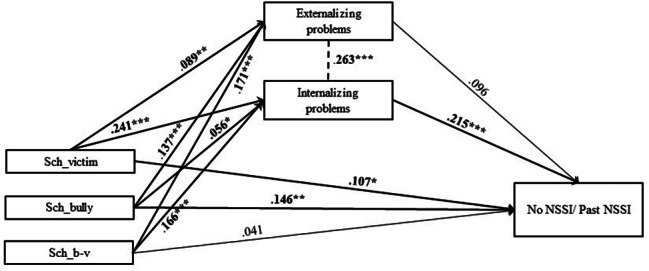
Final Model 1 of school bullying and No/Past NSSI showing standardized coefficients Note: Significant paths are marked with bold numbers and arrows. Sch_victim = school bullying victim; Sch_bully = school bullying perpetrator/bully; Sch_b-v = school bullying bully-victim; *p < .05; **p < .01; ***p < .001

In Model 1, explained variance of past NSSI was 14.8% (Table S1, Table S2, Fig. 7). Despite significant associations in the model, only two significant indirect effect size estimates were presented in this mediation model, suggesting significant mediated pathways: path 1.4. victim – internalizing problems – past NSSI (β = 0.052; SE = 0.02; p < .01); path 1.6. bully-victim – internalizing problems – past NSSI (β = 0.036; SE = 0.01; p < .01) (Table S1, Table S2).

**Fig. 8:**
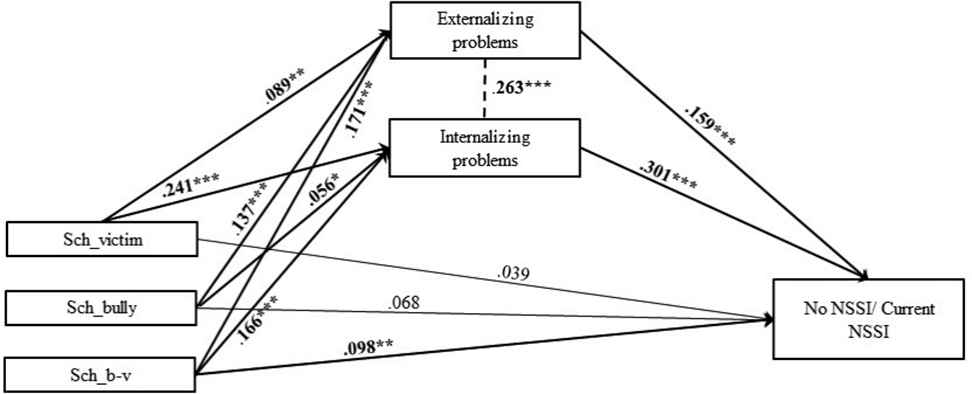
Final Model 2 of school bullying and No/Current NSSI showing standardized coefficients and standard errors Note: Significant paths are marked with bold numbers and arrows. Sch_victim = school bullying victim; Sch_bully = school bullying perpetrator/bully; Sch_b-v = school bullying bully-victim; *p < .05; **p < .01; ***p < .001

In Model 2, explained variance of current NSSI was 18.9% (Table S1, Table S2, Fig. 8). Five significant indirect effect size estimates were presented in this mediation model, suggesting significant mediated pathways: path 2.1. bully – externalizing problems – current NSSI (β = 0.022; SE = 0.01; p < .01); path 2.3. victim – externalizing problems – current NSSI (β = 0.014; SE = 0.01; p < .05); path 2.4. victim – internalizing problems – current NSSI (β = 0.072; SE = 0.01; p < .001); path 2.5. bully-victim – externalizing problems – current NSSI (β = 0.027; SE = 0.01; p < .01); path 2.6. bully-victim – internalizing problems – current NSSI (β = 0.050; SE = 0.01; p < .001) (Table S1, Table S2).

**Fig. 9:**
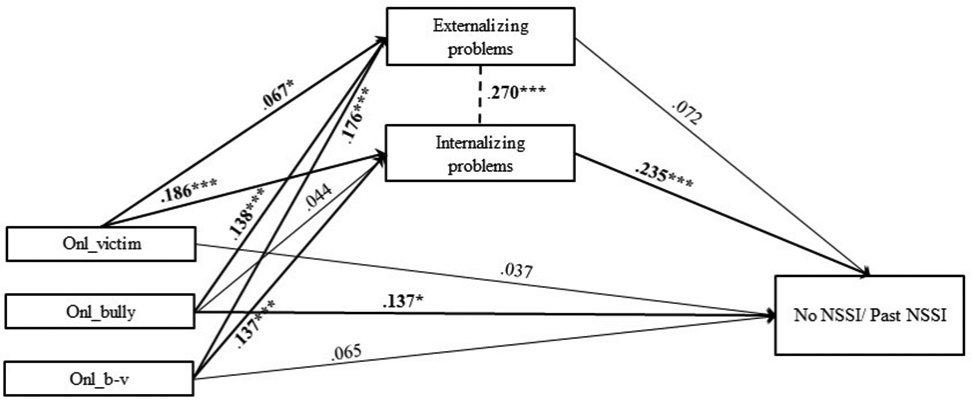
Final Model 3 of online bullying and No/Past NSSI showing standardized coefficients and standard errors Note: Significant paths are marked with bold numbers and arrows. Onl_victim = online bullying victim; Onl_bully = online bullying perpetrator; Onl_b-v = online bullying bully-victim; *p < .05; **p < .01; ***p < .001

In Model 3, explained variance of past NSSI was 13.6% (Table S1, Table S2, Fig. 9). Two significant indirect effect size estimates were presented in this mediation model, suggesting significant mediated pathways: path 3.4. victim – internalizing problems – past NSSI (β = 0.044; SE = 0.01; p < .01); 3.6. bully-victim – internalizing problems – past NSSI (β = 0.032; SE = 0.01; p < .01) (Table S1, Table S2).

**Fig. 10:**
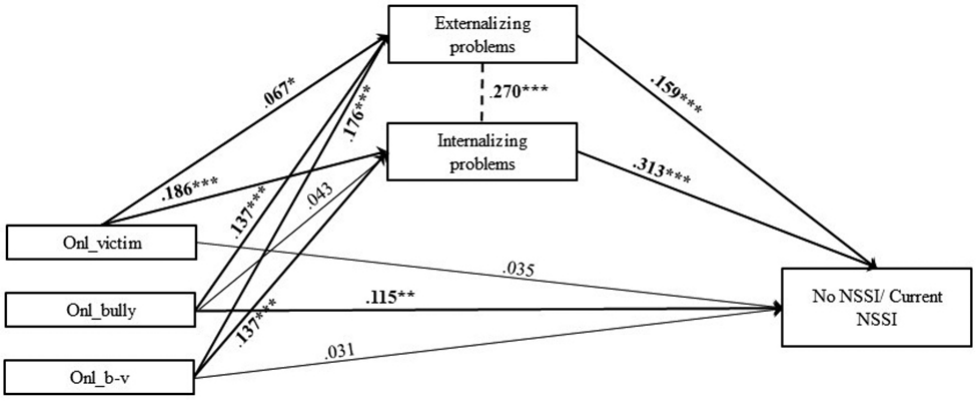
Final Model 4 of online bullying and No/Current NSSI showing standardized coefficients and standard errors Note: Significant paths are marked with bold numbers and arrows. Onl_victim = online bullying victim; Onl_bully = online bullying perpetrator; Onl_b-v = online bullying bully-victim; *p < .05; **p < .01; ***p < .001

In model 4, explained variance of current NSSI was 19.0% (Table S1, Table S2, Fig. 10). Five significant indirect effect size estimates were presented in this mediation model, suggesting significant mediated pathways: path 4.1. bully – externalizing problems – current NSSI (β = 0.022; SE = 0.01; p < .01); path 4.3. victim – externalizing problems – current NSSI (β = 0.011; SE = 0.01; p < .05); path 4.4. victim – internalizing problems – current NSSI (β = 0.058; SE = 0.04; p < .001); path 4.5. bully-victim – externalizing problems – current NSSI (β = 0.028; SE = 0.01; p < .01); path 4.6. bully-victim – internalizing problems – current NSSI (β = 0.043; SE = 0.01; p < .001) (Table S1, Table S2).

**Fig. 11:**
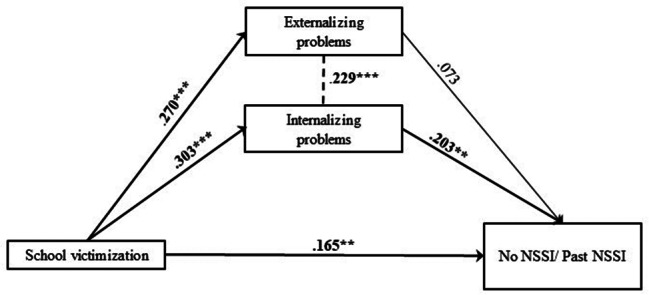
Final Model 5 of school victimization and No/Past NSSI showing standardized coefficients and standard errors Note: Significant paths are marked with bold numbers and arrows. *p < .05; **p < .01; ***p < .001

In Model 5, explained variance of past NSSI was 14.1% (Table S1, Table S2, Fig. 11). One significant indirect effect size estimate was presented in this mediation model, suggesting a significant mediated pathway: path 5.2. school victimization – internalizing problems – past NSSI (β = 0.062; SE = 0.02; p < .01) (Table S1, Table S2).

**Fig. 12:**
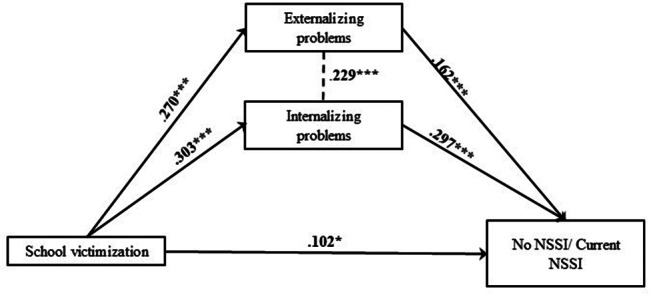
Final Model 6 of school victimization and No/Current NSSI showing standardized coefficients and standard errors Note: Significant paths are marked with bold numbers and arrows. *p < .05; **p < .01; ***p < .001

In Model 6, explained variance of current NSSI was 18.7% (Fig. 12). Two significant indirect effect size estimates were presented in this mediation model, suggesting significant mediated pathways: path (6.1) school victimization – externalizing problems – current NSSI (β = 0.044; SE = 0.01; p < .001); path (6.2) school victimization – internalizing problems – current NSSI (β = 0.090; SE = 0.04; p < .001) (Table S1, Table S2).

## Discussion

Our results indicate that mental health problems and NSSI are significantly more relevant for students who are involved in any form of bullying, either in school or online settings, although differences can be detected between various bullying roles. Internalizing and externalizing problems were significant mediators between different bullying roles and current NSSI, although not in the case of NSSI that occurred in the past. School and online bullying did not differ in significant mediation paths.

Our results show a high level of lifetime NSSI (41.17%) in a high-school student sample, which is similar to the latest NSSI research findings [12].

In our study, we found that more students were involved in traditional bullying than in cyberbullying (23.35% of the students were involved in traditional, school-based bullying, and 16.84% of children were involved in cyberbullying) which is in accordance with similar studies [86]. The greatest difference in the number of students involved in different roles occurs among online (3.05%) and school (7.98%) bullies. It might be because students do not consider their acts as harmful in the online space and the feedback of the victim is not as direct or visible as in a face-to face situation.

Gender differences we found were in line with previously reported results [20, 22]: more girls were victims, more boys were bullies and bully-victims compared to girls, both in school and in online settings. Feijóo and her colleagues [87] – measuring school victim status in two ways – found that boys suffered more physical violence, were insulted, called names and were threatened, while girls were victims of more relational bullying behaviors (e.g., were excluded or ignored; had rumors spread about them).

Our results show that when measuring concrete types of school victimization, remarkably more students report being victims compared to when only the frequency of being a victim is measured without specific types of bullying listed. It raises attention on the possible phenomenon that high-school students might not be familiar with the terms of assault and victimization. It is also possible that they are not aware of the fact that certain behaviors towards them in school settings are considered as intentional harm doing or aggression. This result is a valuable information for teachers and scientists: it suggests that students might not be aware of the concept of bullying or that different harmful acts should be considered peer violence.

In accordance with meta-analytical findings [40], victims, bullies and bully-victims were more likely to engage in NSSI than their peers who were not involved in bullying. Students involved in NSSI report more frequent school victimization compared to peers not involved in bullying. Furthermore, those who were not involved in any role of bullying (neither at school or online) reported using significantly less NSSI methods compared to involved participants. This can be interpreted by using the interpersonal theory of NSSI [88], which considers NSSI as a negative coping strategy, aimed to reduce the stress caused by negative interpersonal events, such as bullying. Bullying is an adverse interpersonal event as a victim, but also as a perpetrator [40]. Furthermore, the General Strain Theory [89] suggests that bullying can be experienced as an unjust act that can be resolved with an aggressive behavior. From the victim’s perspective, aggression towards oneself might be the only available option, thus self-harm can be perceived as a temporarily effective way to manage one’s own stress [40].

Students who are involved in school or online bullying use more NSSI methods than not involved peers, and among them, bully-victims use the most. The number of NSSI episodes (e.g., how often it occurs) in our study did not differentiate between students who are involved and who are not involved in bullying. Although many articles use the frequency of NSSI episodes as an indicator of NSSI severity [57], it is suggested that the number of NSSI methods predict severity more significantly. NSSI frequency and the number of used methods can also interact, defining a subgroup of individuals seriously at risk [90]. Robinson and colleagues [91] found in a community adolescent sample that among adolescents with a lifetime history of NSSI, the number of NSSI methods was strongly associated with reporting suicidal thoughts and behaviors while the number of NSSI episodes was not.

Those who currently engage in NSSI seem to be vulnerable for both externalizing and internalizing problems. Bully-victims reported the highest level of externalizing symptoms in school and online settings, while victims’ level of internalizing problems was the highest both in school and online settings. Higher levels of internalizing problems were present in bully-victims as well. Internalizing symptoms are often conceptualized as significant negative longitudinal outcomes of bullying victimization [92], however this longitudinal association seem to be bidirectional [93]. Peer victimization – considered as a significant stressor – can result in internalizing symptoms in adolescents who tend to interpret stressful events in a self-critical manner [32]. Furthermore, internalizing problems might increase the risk of becoming a target of peer victimization due to individual vulnerabilities (e.g., social withdrawal, avoidance, fearfulness) [94]. A result that might raise attention on the possible differences of the nature of online and school bullying is that bullies and bully-victims reported higher level of externalizing problems in online settings than in school settings. Online bully-victims also reported significantly higher levels of externalizing symptoms than online victims, a difference, which was not present in school settings. A possible explanation of this might be the online disinhibition phenomenon [95], that suggests that in online settings users tend to lose their normal capacity of inhibition, partly or completely, as there is no fear of rejection or judgement [96]. Online bullying might also be a more impulsive act, as the perpetrator has no personal connection with the victim, no facial expression of the victim’s emotions is available and no acquaintance, previous personal contact or physical imbalance is needed [86, 97].

In our study we found that, externalizing and internalizing symptoms are more present in students involved in any role of bullying compared to their not involved peers, but different roles seem to be associated differently to symptoms. The differences were not always significant between bullying roles: the results suggest that bully-victims are the most vulnerable group in school and online bullying regarding mental health problems, both in externalizing and internalizing problems. It might be because bully-victims are rejected and isolated by peers and at the same time they are influenced negatively (e.g., to engage in fights) by those adolescents they are friends with [29]. This suggests that contextual predictors (e.g., peer status and peer influence) can be essential to deal with the bully-victim status. In accordance with Cook’s [29] meta-analytical findings our results suggest that a bully is possibly an adolescent with significant externalizing behaviors, and also having internalizing symptoms. A victim is an adolescent showing major internalizing symptoms but also engaging in externalizing behaviors to some extent. A bully-victim possibly has comorbid externalizing and internalizing problems which can further worsen his or her mental health.

Models including current NSSI show slightly higher explained variances than those investigating past NSSI. Regarding the mediation models, our main question was whether externalizing and internalizing symptoms mediate the association between different school and online bullying roles and current and past NSSI. Based on indirect effects, results show diverse mediation patterns with specific paths identified regarding different bullying roles.

When NSSI occurred in the past but not currently, both online and school victim and bully-victim roles were significantly associated to NSSI via internalizing problems. The results also underline that school and online victim roles are more strongly associated to internalizing problems and suggest that bully-victims might have comorbid internalizing and externalizing symptoms. Only internalizing symptoms emerged as significant mediators due to the lack of association between externalizing symptoms and past NSSI that happened at least a month before data collection. Based on our mediation analysis and other results, internalizing symptoms are more strongly associated to NSSI (both past and current) than externalizing problems. Emotional and internalizing disorders show clear conceptual overlap with NSSI, as in emotional disorders, negative emotions are often experienced (e.g., fear, anxiety, sadness), which can possibly be maintained by a maladaptive avoidant or coping strategy, like NSSI [98]. Although, a systematic review suggests that externalizing pathology is also strongly associated to self-injurious behaviors [56], the study included a wide range of externalizing problems (e.g., attention deficit hyperactivity disorder, oppositional defiant disorder, intermittent explosive disorder) that our study did not, furthermore they included studies which did not differentiate between nonsuicidal and suicidal self-injury. Some studies found a link between externalizing pathology and NSSI happened in the previous year (e.g., [57], but in our study, past NSSI could occur any time earlier in life, therefore, developmental aspect might play a role in the association of NSSI and externalizing symptoms. Furthermore, the questionnaire asked about externalizing and internalizing symptoms in the previous 6 months, but past NSSI could have occurred earlier than that.

In models with current NSSI, externalizing and internalizing problems seem to be a considerable and significant mediator at most of the bullying roles both in school and online settings. Only bully role was not associated to current NSSI via internalizing problems, which is in accordance with the study’s previous findings, namely that bully role is strongly associated to externalizing problems. Victim and bully-victim status were both associated to current NSSI via externalizing and internalizing symptoms as well, which suggests that not only bully-victims might show comorbid internalizing and externalizing symptoms [29], but victims as well. Therefore, future research should put special attention on bully-victims and also on victims to specify which leading symptom(s) might be in direct association to the involvement in bullying. Longitudinal studies can reveal the dynamics of the development of being a bully-victim: whether bully-victims were victimized first (i.e., bullied by others) and then started to bully others, or in the opposite way, whether they were initially bullies who then became victims because others took revenge against them [44, 99]. A study found that victims in a bullying episode might use aggressive strategies to cope with the situation that tend to perpetuate and escalate the bullying interaction [100] and therefore might make them a bully-victim. This might be especially true for victims with a relatively high level of externalization [86] which can also explain our findings that victim status was associated not only to internalizing but to externalizing symptoms as well. In the bully-victim role, guilt might have a special role as well due to the experiences both as a perpetrator who commits the same acts as were done to the person previously [101].

The results of the models containing school and online bullying, victim role was confirmed by the last two models, containing the frequency of different school victimization types; frequency of school victimization was associated to past NSSI only via internalizing symptoms, while to current NSSI both types of symptoms emerged as significant paths.

The mediation analysis, the settings of bullying (school or online) did not show differences regarding the significant paths via the mediators, which indicates that, in the association of bullying and NSSI, internalizing and externalizing symptoms do not differentiate between school and online settings. The results also suggest that internalizing and externalizing symptoms should be addressed when NSSI occurs currently in a student’s life. As externalizing and internalizing problems only partly mediate the association between different bullying roles and NSSI, to build a complex model, other factors should be considered as well. Some psychological features had been already identified as mediator variables, like social self-efficacy (an individual’s belief that he or she can effectively carry out social tasks) [47, 102], negative emotions [45], depressive mood and depressive symptoms [22, 37]. It is also essential to identify factors that can help to cope with stress due to bullying and therefore prevent NSSI as a possible maladaptive coping strategy. Hay and Meldrum [45] found that the relationship between bullying victimization and NSSI almost disappeared in those adolescents who experienced supportive parenting practices. The need for evidence-based guidelines to prevent and react to NSSI behaviors within schools had already been articulated [103] and the current study emphasizes its importance by highlighting that school-related factors, like bullying, is associated to NSSI.

Finally, limitations of this study are considered. A possible limitation of the study is its cross-sectional nature, which does not allow any assumptions, whether bullying or externalizing and internalizing problems are predicting NSSI or not. Another limitation might be the validity of the measurement of different bullying roles. In the current study, when asking the frequency of concrete school victimization types, participants reported a remarkably higher occurrence of school victimization than when asking only the frequency in general. As different bullying roles (victim, bully, bully-victim) were established based on the reported frequency (without asking concrete acts), it is possible that participants would have reported a higher and therefore more valid frequency of bully acts based on different types of bully acts given. This limitation however raises attention to the importance of making awareness of the concept and nature of bullying and peer violence in schools. A relatively high prevalence of bullying might be because one single act of bullying (perpetration, victimization, or both) was enough to fulfill a certain category of bullying role. Regarding bullying roles, another limitation should be the possible clustering effect of students from the same class (students from the same class know each other and spend a lot of time together), that was not controlled in the current study. Future studies using more robust analyses (e.g., multilevel structural equation modeling) are suggested to take care of this statistical issue. The unequal number of male and female participants in this study should be considered a limitation, as gender differences are remarkable in NSSI [104] and in bullying [24] as well.

In our study, we applied a traditional way of distinguishing different bullying roles (bully, victim, bully-victim) [19, 20] however, according to other perspectives, children could fall along a bully-victim continuum and roles demonstrate a significant overlap [105]. The results should be interpreted with the approach that due to the possible overlap between different bullying roles that were not taken into consideration in the current study, it is possible that students involved in both online and school bullying but in different roles have different psychological needs and difficulties compared to students being involved in one form of bullying, in one single role. Therefore, in future studies, latent cluster or latent profile analyses should be applied to be able to distinguish these, often co-occurring bullying roles.

Although, the sample size of the current study is adequate to make complex statistical analyses, eight subgroups were formed (school victims, school bullies, school bully-victims, not involved participants in school bullying, online victims, online bullies, online bully-victims, not involved participants in online bullying) from which the group of online bullies contains only n = 31 participants.

Current NSSI seems to be more relevant regarding bullying in our study, but a limitation might be that past NSSI could have occurred any time during life, while bullying roles and psychological symptoms (externalizing and internalizing problems) were measured based on the occurrence during the previous few months, or previous six months, respectively. Finally, regarding that our study focused exclusively on the path through which bullying is linked to NSSI via externalizing and internalizing symptoms, future research should focus on other possible mediator and moderator variables.

## Conclusions

Based on the results, students involved in bullying are more vulnerable to NSSI and to psychological symptoms compared to their peers who are not involved in bullying. Externalizing and internalizing problems do significantly mediate the association of different bullying roles and NSSI, but to different extent and through different paths. Psychological symptoms seem to play a significant role when NSSI occurs currently throughout the last month. Bully role seems to be associated firstly to externalizing symptoms, but internalizing problems can be present as well. Victim role seems to be slightly associated to externalizing problems, but internalizing symptoms should be addressed in the first place. At bully-victims, comorbid internalizing and externalizing symptoms might occur, however their engagement in NSSI does not seem to be more severe than victims’ or bully’s engagement. Bullying prevention is important because its connection to NSSI is significant. Inconsistencies regarding the self-report of victim role and different types of victimization raises attention on the importance of raising awareness on the phenomenon of bullying and empowering more vulnerable students to be conscious about being maltreated by peers.

### Electronic supplementary material

Below is the link to the electronic supplementary material.

**Supplementary Material 1:** Table S1 Standardized regression coefficients and total, direct, and indirect effects related to each model.

**Supplementary Material 2:** Table S2 Odds ratios and 95% confidence intervals of associations between independent, mediator and outcome variables.

## Data Availability

The datasets used and/or analyzed during the current study are available from the corresponding author on reasonable request.

## Abbreviations

NSSI: Nonsuicidal self-injury
WHO: World Health Organization
HBSC study: Health Behaviour in School-aged Children study
ISAS: Inventory of Statements About Self-Injury
SDQ: Strengths and Difficulties Questionnaire
SD: Standard deviation
