# Supplementary material for: Online and school bullying roles: are bully-victims more vulnerable in nonsuicidal self-injury and in psychological symptoms than bullies and victims?
Source: BMC Psychiatry. 2023 Dec 14;23:945. doi: 10.1186/s12888-023-05341-3 (PMC10722836; doi:10.1186/s12888-023-05341-3)
Supplement: Supplementary file 1 — Supplementary Material 1: Table S1 Standardized regression coefficients and total, direct, and indirect effects related to each model. [file 12888_2023_5341_MOESM1_ESM.docx]

| Table S1  Standardized regression coefficients and total, direct, and indirect effects related to each model. | | | | | | | | | | | | |
| --- | --- | --- | --- | --- | --- | --- | --- | --- | --- | --- | --- | --- |
|  | Model parameters | | | | | |  | | *Effects for latent response variable* | | | |
| Models | *Path a*  β (S.E.) | *Path b*  β (S.E.) | *Path c’* β (S.E.) | *R^2^* mediation | *R^2^* outcome |  | | SIE β (S.E.) | | TIE β  (S.E.) | DE β  (S.E.) | TE β  (S.E.) |
| *1. School bullying roles – Past NSSI* | | |  | R^2^ exter. = 5.0%  R^2^ inter. = 17.0% | 14.8% |  | |  | |  |  |  |
| - 1. bully – externalizing – past NSSI | **0.137*****  **(0.03)** | 0.096  (0.06) | **0.146****  **(0.05)** |  |  |  | | 0.013  (0.01) | | **0.025***  **(0.01)** | **0.146****  **(0.05)** | **0.171****  **(0.05)** |
| - 1. bully – internalizing – past NSSI | **0.056***  (0.06) | **0.215*****  **(0.07)** | **0.146****  **(0.05)** |  |  |  | | 0.012  (0.01) | |  |  |  |
| - 1. victim – externalizing – past NSSI | **0.089****  **(0.03)** | 0.096  (0.06) | **0.107***  **(0.05)** |  |  |  | | 0.009  (0.01) | | **0.060****  **(0.02)** | **0.107***  **(0.05)** | **0.167****  **(0.05)** |
| - 1. victim – internalizing – past NSSI | **0.241*****  **(0.03)** | **0.215*****  **(0.07)** | **0.107***  **(0.05)** |  |  |  | | **0.052****  **(0.02)** | |  |  |  |
| - 1. bully-victim – externalizing – past NSSI | **0.171*****  **(0.03)** | 0.096  (0.06) | 0.041  (0.07) |  |  |  | | 0.016  (0.01) | | **0.052** (0.01)** | 0.041  (0.07) | 0.093  (0.07) |
| - 1. bully-victim – internalizing – past NSSI | **0.166*****  **(0.03)** | **0.215*****  **(0.07)** | 0.041  (0.07) |  |  |  | | **0.036****  **(0.01)** | |  |  |  |
| 1. *School bullying roles – Current NSSI* | | |  | R^2^ exter. = 5.0%  R^2^ inter. = 17.0% | 18.9% |  | |  | |  |  |  |
| - 1. bully – externalizing – current NSSI | **0.137*****  **(0.03)** | **0.159*****  **(0.04)** | 0.068  (0.04) |  |  |  | | **0.022****  **(0.01)** | | **0.039****  **(0.01)** | 0.068  (0.04) | **0.107****  **(0.04)** |
| - 1. bully – internalizing – current NSSI | **0.056***  **(0.03)** | **0.301*****  **(0.04)** | 0.068  (0.04) |  |  |  | | 0.017  (0.01) | |  |  |  |
| - 1. victim – externalizing – current NSSI | **0.089****  **(0.03)** | **0.159*****  **(0.04)** | 0.039  (0.04) |  |  |  | | **0.014***  **(0.01)** | | **0.087*****  **(0.01)** | 0.039  (0.04) | **0.126****  **(0.04)** |
| - 1. victim – internalizing – current NSSI | **0.241*****  **(0.03)** | **0.301*****  **(0.04)** | 0.039  (0.04) |  |  |  | | **0.072*****  **(0.01)** | |  |  |  |
| - 1. bully-victim – externalizing – current NSSI | **0.171*****  **(0.03)** | **0.159*****  **(0.04)** | **0.098****  **(0.04)** |  |  |  | | **0.027****  **(0.01)** | | **0.077*****  **(0.01)** | **0.098****  **(0.04)** | **0.176*****  **(0.04)** |
| - 1. bully-victim – internalizing – current NSSI | **0.166*****  **(0.03)** | **0.301*****  **(0.04)** | **0.098****  **(0.04)** |  |  |  | | **0.050*****  **(0.01)** | |  |  |  |
| 1. *Online bullying roles – Past NSSI* | | |  | R^2^ exter. = 5.2%  R^2^ inter. = 14.2% | 13.6% |  | |  | |  |  |  |
| - 1. bully – externalizing – past NSSI | **0.138*****  **(0.03)** | 0.072  (0.06) | **0.137***  **(0.06)** |  |  |  | | 0.010  (0.01) | | 0.020  (0.01) | **0.137***  **(0.06)** | **0.157****  **(0.06)** |
| - 1. bully – internalizing – past NSSI | 0.044  (0.03) | **0.235*****  **(0.06)** | **0.137***  **(0.06)** |  |  |  | | 0.010  (0.01) | |  |  |  |
| - 1. victim – externalizing – past NSSI | **0.067***  **(0.03)** | 0.072  (0.06) | 0.037  (0.06) |  |  |  | | 0.05  (0.01) | | **0.049****  **(0.02)** | 0.037  (0.06) | 0.085  (0.06) |
| - 1. victim – internalizing – past NSSI | **0.186*****  **(0.03)** | **0.235*****  **(0.06)** | 0.037  (0.06) |  |  |  | | **0.044****  **(0.01)** | |  |  |  |
| - 1. bully-victim – externalizing – past NSSI | **0.176*****  **(0.04)** | 0.072  (0.06) | 0.065  (0.05) |  |  |  | | 0.013  (0.01) | | **0.045****  **(0.02)** | 0.065  (0.05) | **0.110***  **(0.05)** |
| - 1. bully-victim – internalizing – past NSSI | **0.137*****  **(0.03)** | **0.235*****  **(0.06)** | 0.065  (0.05) |  |  |  | | **0.032****  **(0.01)** | |  |  |  |
| 1. *Online bullying roles – Current NSSI* | | |  | R^2^ exter. = 5.2%  R^2^ inter. = 14.2% | 19.0% |  | |  | |  |  |  |
| - 1. bully – externalizing – past NSSI | **0.137*****  **(0.03)** | **0.159*****  **(0.04)** | **0.115****  **(0.04)** |  |  |  | | **0.022****  **(0.01)** | | **0.035****  **(0.01)** | **0.115****  **(0.04)** | **0.150*****  **(0.04)** |
| - 1. bully – internalizing – past NSSI | 0.043  (0.03) | **0.313*****  **(0.04)** | **0.115****  **(0.04)** |  |  |  | | 0.013  (0.01) | |  |  |  |
| - 1. victim – externalizing – past NSSI | **0.067***  **(0.03)** | **0.159*****  **(0.04)** | 0.035  (0.04) |  |  |  | | **0.011***  **(0.01)** | | **0.069*****  **(0.01)** | 0.035  (0.04) | **0.104****  **(0.04)** |
| - 1. victim – internalizing – past NSSI | **0.186*****  **(0.03)** | **0.313*****  **(0.04)** | 0.035  (0.04) |  |  |  | | **0.058*****  **(0.04)** | |  |  |  |
| - 1. bully-victim – externalizing – past NSSI | **0.176*****  **(0.04)** | **0.159*****  **(0.04)** | 0.031  (0.04) |  |  |  | | **0.028****  **(0.01)** | | **0.071*****  **(0.01)** | 0.031  (0.04) | **0.102****  **(0.04)** |
| - 1. bully-victim – internalizing – past NSSI | **0.137*****  **(0.03)** | **0.313*****  **(0.04)** | 0.031  (0.04) |  |  |  | | **0.043*****  **(0.01)** | |  |  |  |
| 1. *School victimization – Past NSSI* | | |  | R^2^ exter. = 7.5%  R^2^ inter. = 18.4% | 14.1% |  | |  | |  |  |  |
| - 1. school vict. – externalizing – past NSSI | **0.270*****  **(0.03)** | 0.073  (0.06) | **0.165****  **(0.05)** |  |  |  | | 0.020  (0.02) | | **0.082****  **(0.02)** | **0.165****  **(0.05)** | **0.247*****  **(0.05)** |
| - 1. school vict. – internalizing – past NSSI | **0.303*****  **(0.03)** | **0.203****  **(0.06)** | **0.165****  **(0.05)** |  |  |  | | **0.062****  **(0.02)** | |  |  |  |
| 1. *School victimization – Current NSSI* | | |  | R^2^ exter. = 7.5%  R^2^ inter. = 18.4% | 18.7% |  | |  | |  |  |  |
| - 1. school vict. – externalizing – current NSSI | **0.270*****  **(0.03)** | **0.162*****  **(0.04)** | **0.102***  **(0.04)** |  |  |  | | **0.044*****  **(0.01)** | | **0.134*****  **(0.02)** | **0.102***  **(0.04)** | **0.236*****  **(0.04)** |
| - 1. school vict. – internalizing – current NSSI | **0.303*****  **(0.03)** | **0.297*****  **(0.04)** | **0.102***  **(0.04)** |  |  |  | | **0.090*****  **(0.04)** | |  |  |  |
| *Note. Path a* represents the relationship between independent and mediator variables. *Path b* represents the relationship between mediator variables and categorical outcome variable. *Path c’* represents the relationship between independent variables and categorical outcome variable while taking into account the effect of the mediator variable. Standardized effect size values displayed with bold figures are significant. School vict. = school victimization; S.E. = standard error; *p<0.05; **p<0.01; ***p<0.001; R^2^mediator: explained variance related to the mediator variable; R^2^exter. = explained variance of externalizing problems; R^2^inter. = explained variance of internalizing problems; R^2^outcome: explained variance related to the outcome variable; SIE = specific indirect effect; TIE = total indirect effect; DE = direct effect; TE = total effect. | | | | | | | | | | | | |
