# Supplementary material for: Online and school bullying roles: are bully-victims more vulnerable in nonsuicidal self-injury and in psychological symptoms than bullies and victims?
Source: BMC Psychiatry. 2023 Dec 14;23:945. doi: 10.1186/s12888-023-05341-3 (PMC10722836; doi:10.1186/s12888-023-05341-3)
Supplement: Supplementary file 2 — Supplementary Material 2: Table S2 Odds ratios and 95% confidence intervals of associations between independent, mediator and outcome variables. [file 12888_2023_5341_MOESM2_ESM.docx]

| Table S2  Odds ratios and 95% confidence intervals of associations between independent, mediator and outcome variables | | | | | | | | |  |  |  |
| --- | --- | --- | --- | --- | --- | --- | --- | --- | --- | --- | --- |
|  |  | Past NSSI | | | | Current NSSI | | | | |  |
|  | OR | 95% CI  [LL; UL] | p |  | OR | | 95% CI  [LL; UL] | p | |  |  |
| *School bullying roles* | | | | | | | | | | | |
| Bully role | 2.86 | [1.34; 6.10] | .006 |  | 1.66 | | [0.98; 2.80] | .057 | |  |  |
| Victim role | 2.09 | [1.01; 4.33] | .047 |  | 1.32 | | [0.79; 2.18] | .279 | |  |  |
| Bully-victim role | 1.40 | [0.44; 4.39] | .567 |  | 2.29 | | [1.23; 4.28] | .009 | |  |  |
| Externalizing problems | 1.06 | [0.98; 1.15] | .114 |  | 1.11 | | [1.05; 1.17] | <.001 | |  |  |
| Internalizing problems | 1.12 | [1.04; 1.20] | .001 |  | 1.18 | | [1.12; 1.23] | <.001 | |  |  |
| Age | 1.29 | [1.09; 1.53] | .002 |  | 0.95 | | [0.85; 1.06] | .358 | |  |  |
| Gender | 0.85 | [0.51; 1.44] | .548 |  | 1.32 | | [0.93; 1.88] | .119 | |  |  |
| *Online bullying roles* | | | | | | | | | | | |
| Bully role | 4.69 | [1.74; 18.75] | .026 |  | 3.81 | | [1.52; 9.55] | .004 | |  |  |
| Victim role | 1.28 | [0.57; 2.91] | .550 |  | 1.28 | | [0.76; 2.17] | .356 | |  |  |
| Bully-victim role | 1.85 | [0.66; 5.16] | .239 |  | 1.35 | | [0.66; 2.77] | .411 | |  |  |
| Externalizing problems | 1.05 | [0.97; 1.13] | .254 |  | 1.11 | | [1.05; 1.17] | <.001 | |  |  |
| Internalizing problems | 1.13 | [1.05; 1.21] | <.001 |  | 1.18 | | [1.13; 1.24] | <.001 | |  |  |
| Age | 1.29 | [1.09; 1.53] | .002 |  | 0.95 | | [0.85; 1.05] | .318 | |  |  |
| Gender | 0.83 | [0.50; 1.40] | .491 |  | 1.26 | | [0.89; 1.78] | .196 | |  |  |
| *School victimization* | | | | | | | | | | | |
| School victimization | 1.13 | [1.04; 1.22] | .002 |  | 1.08 | | [1.01; 1.15] | .018 | |  |  |
| Externalizing problems | 1.05 | [0.97; 1.13] | .225 |  | 1.11 | | [1.06; 1.17] | <.001 | |  |  |
| Internalizing problems | 1.11 | [1.04; 1.19] | .002 |  | 1.17 | | [1.12; 1.23] | <.001 | |  |  |
| Age | 1.30 | [1.10; 1.54] | .002 |  | 0.95 | | [0.85; 1.06] | .369 | |  |  |
| Gender | 0.80 | [0.48; 1.34] | .392 |  | 1.25 | | [0.88; 1.77] | .208 | |  |  |
| *Note*. OR = odds ratio; CI = confidence interval; LL and UL indicate the lower and upper limits of a confidence interval, respectively. | | | | | | | | | | | |
